# Assessment of Safety Profile of Activated Curcumin C3 Complex (AC^3^®), Enriched Extract of Bisdemethoxycurcumin from the Rhizomes of *Curcuma longa*

**DOI:** 10.1155/2023/3729399

**Published:** 2023-10-31

**Authors:** Muhammed Majeed, Sarang Bani, Anjali Pandey, Muhamad Ibrahim A., Smitha Thazhathidath

**Affiliations:** ^1^Sami-Sabinsa Group Limited, 19/1 & 19/2, I Main, II Phase, Peenya Industrial Area, Bengaluru, Karnataka 560058, India; ^2^Sabinsa Corporation, 20 Lake Drive, East Windsor 08520, NJ, USA

## Abstract

The present work was carried out to investigate the toxic effects of Activated Curcumin C3 Complex (AC^3^®) through the methods of acute, subacute, subchronic, reproductive/developmental toxicity, and genotoxicity when administered orally in experimental rodents. The studies were carried out in line with OECD principles of good laboratory practice. A single-dose acute oral toxicity study was conducted on female Wistar rats that produced no toxic effects after 14 days (the observation period) of treatment. Subacute, subchronic, and reproductive/developmental studies were conducted in Wistar rats, divided equally into vehicle control, 125, 250, and 500 mg/kg dose groups along with recovery groups for vehicle control and high dose. In all the studies, there were no abnormal clinical signs/behavioral changes, reproductive and developmental parameters, or gross and histopathological changes. Likewise, no alteration was found in the body weight, hematology, and other biochemical parameters. Also, it did not show mutagenicity in the *in vitro* AMES test or clastogenicity and aneugenicity in the *in vivo* micronucleus test, indicating that AC^3^® did not induce any genotoxic effects. This revealed that oral administration of AC^3^® is safe in rodents, nonmutagenic, and had no observed adverse effects under experimental conditions.

## 1. Introduction

**Figure 1:**
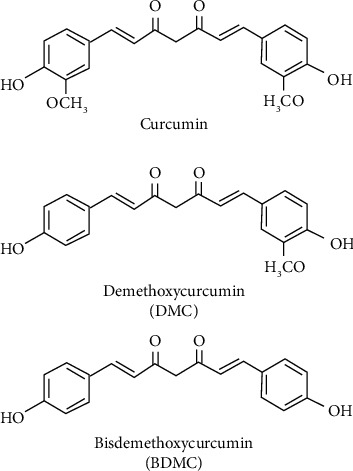
Structure of curcumin, bisdemethoxycurcumin, and demethoxycurcumin.

Turmeric the “golden spice” or “spice of life” derives its Latin name, *Curcuma longa*, from the Arabic name, “Kurkum”—Turmeric native to Indian subcontinent has held its supreme position since Vedic period―owing to its wide array of utilities. Several traditional healing systems, including Ayurveda have been known to use turmeric in managing various conditions, including those of the skin, pulmonary, and gastrointestinal systems, diabetes, wounds, liver disorders, and inflammatory conditions [1]. The compounds obtained from *Curcuma* species, specifically curcuminoids, and their role in the therapeutic management of various diseases and disorders is well documented [2]. The metabolites of curcumin are garnering the attention of researchers owing to their similar or superior efficacy of curcumin [3]. In general, extracts of turmeric are dominated by the presence of curcumin (75–81%), followed by demethoxycurcumin (DMC) (15–19%) and bisdemethoxycurcumin (BDMC) (2.2–6.5%). It is believed that a mixture of phytonutrients consisting of curcumin and two naturally cooccurring DMC and BDMC shows better pharmacological efficacy than curcumin alone (Figure 1).

Activated Curcumin C3 Complex (AC^3^®) is an exemplary formulation of BDMC-enriched *C. longa* extract, which possesses improved physiological benefits compared to regular turmeric extract. Removal of methoxy groups from curcumin basic structure yields DMC and BDMC, which are different, many times superior, from the parent molecule in their chemical and biological properties. The synergistic and complementary actions of these bioactive molecules provide better efficacy in several aspects of pharmacological effects. Various *in vivo* and *in vitro* studies have shown that BDMC is more bioaccessible and bioavailable than its parent curcumin. Studies have shown that BDMC is well documented as a chemopreventive and antitumor agent that can modulate several molecular pathways and cellular targets involved in different stages of tumor initiation, growth, and metastasis [4–8]. BDMC has been reported to be more potent than curcumin in inhibiting NF-*κ*B and did not require oxidative activation as observed for curcumin, suggesting it as a superior anti-inflammatory molecule [9]. It also reduced osteoclast formation and related health complications like osteoporosis *in vivo* [9], possesses hypolipidemic activity as it significantly reduces triglycerides and total cholesterol levels in the serum and the liver [10]. In several *in vitro* and *in vivo* studies, BDMC has been proven for antiulcer [11] and antidiabetic activity, where it has shown better or equal efficacy compared to other curcuminoids [12–14]. In addition, BDMC also inhibited the activity of acetylcholinesterase more efficiently than DMC or curcumin *in vitro* [15]. Thus, BDMC shares several useful pharmacological activities with curcumin [16]. The mechanistic work carried out by several academic groups in conjunction with our work in this area including metabolic health, antiviral, lung and kidney function, and joint health paved the way for the emergence of BDMC, the major constituent in AC^3^®. Irrespective of the various pharmacological properties, there is a few evidence of BDMC for its toxicity data. Numerous preclinical and clinical studies have suggested curcumin is safe and well tolerated. Curcumin has been approved by the United States Food and Drug Administration as being GRAS (generally recognized as safe) food ingredient [17] and as a supplement in several countries [18]. In addition, studies have shown that *Curcuma* species procured from different places showed varied BDMC content [19, 20], which may have an impact on efficacy and safety profile. Thus, the present work was taken up to summarize the toxic effects of AC^3^® using acute, subacute, subchronic, reproductive/developmental, and genotoxicity (bacterial reverse mutation and mammalian bone marrow micronucleus test).

## 2. Materials and Methods

### 2.1. Chemicals

**Figure 2:**
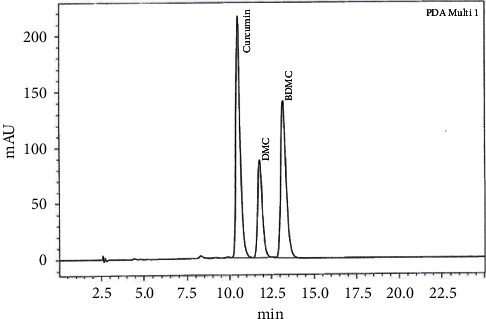
HPLC chromatogram of AC^3^®.

The test sample AC^3^®, an exemplary formulation of BDMC enriched *C. longa* extract, obtained through solvent extraction is standardized to contain a minimum of 85% w/w of total curcuminoids by HPLC. The BDMC content in *Curcuma longa* extract is 2.2 to 6.5% whereas in the test sample AC^3^® is in the range between 30 and 35%. The test sample is prepared by phytochemical department and the reference sample has been stored at Sami-Sabinsa Group Limited, Bengaluru, India. The HPLC chromatogram of the compound at 420 nm is shown in Figure 2.

For bacterial reverse mutation assay, *Salmonella typhimurium* strains (TA98, TA100, TA1535, TA1537, and TA102) and chemicals (sodium azide, 2-nitrofluorene, 9-aminoacridine, mitomycin-C, Benzo[a]pyrene) were used for the study. In addition, 5% and 10% v/v cofactor supplemented postmitochondrial S9 fraction, prepared from rat liver were also used in the study. The chemicals and solvents used throughout the experiments were of analytical grade.

### 2.2. Animals

For acute, subacute, subchronic, and reproductive/developmental toxicity studies Wistar rats were housed in a polypropylene cage with maintained environment of room temperature of 19.1–23.9°C, relative humidity of 45–67.6% with 12 h light and 12 h dark cycle. Animals were fed with a pelleted rodent diet and purified filtered/reverse osmosis (RO) water was provided ad libitum. Animals were kept in sterilized corn cob beddings changed along with the cage twice a week during acclimatization and entire experimental study period.

For genotoxic evaluation, Swiss albino mice were used in mammalian bone marrow micronucleus test. The animals were housed in polycarbonate cages and were maintained at room temperature 20.6°C–22.7°C and relative humidity 53–68% with 12 h light and 12 h dark cycle. A rodent pelleted diet and aquaguard filtered drinking water was offered *ad libitum*.

For acute toxicity and bone marrow micronucleus tests, the animals were obtained from Biogen Laboratory Animal Facility, Bengaluru. For subacute, subchronic, and reproductive/development toxicity studies the animals were obtained from Palamur Biosciences Private Limited, Telangana.

### 2.3. Ethics

The Institutional Animal Ethics Committee (IAEC) of Diligence Bio Private Ltd., Pondicherry (Certificate 121 No. 2067/PO/RcBiBt/S/19/CPCSEA) approved the individual protocol for acute (DB/IAEC/AORT/AG/032/05-21) and bone marrow micronucleus (DB/IAEC/BMNT/AG-008-011/06-20) study. Similarly, Palamur Biosciences Private Limited, Telangana (Certificate No. 1312/PO/RcBiBt-S/RcBiBt-L/09/CPCSEA) approved the individual protocol for subacute (PAL/IAEC/2021/11/01/37), subchronic (PAL/IAEC/2021/01/11/38), and reproductive/developmental (PAL/IAEC/2021/07/01/13) toxicity studies.

The studies were conducted in accordance with the recommendations of the committee for the purpose of control and supervision of experiments on animals (CPCSEA) guidelines for laboratory animal facility published in the Gazette of India, on December 15^th^, 1998. The studies were performed in compliance with OECD principles of Good Laboratory Practice, which ensures and promotes consistency, quality, safety, and reliability during nonclinical and laboratory testing. The guidelines for testing of chemicals no. 423, 407, 408, 421, 471, and 474 were in accordance with the standard operating procedure of the institution.

### 2.4. Acute Oral Toxicity Study

The acute oral toxicity study was conducted as per the OECD guidelines 423 [21]. Healthy young six female adult Wistar rats (3 animals per step) weighing between 175 and 195 g, 10–12 weeks old were selected for the study. All the animals were fasted overnight (feed withheld but with free access to water) prior to dose administration and approximately 3 to 4 hours after dosing. The required quantity of AC^3^® was weighed and then mixed with distilled water. The AC^3^® was administered orally by gavage as single dose using stainless steel ball tipped intubation needle. The dose was adjusted according to the body weight which was measured on the dosing day and dose volume was 10 mL/kg. The dose of 2000 mg/kg body weight (as per OECD guidelines 423, the maximum dose level used in the study) was administered in both Step 1 and Step II animals. Clinical signs of toxicity and mortality were monitored at 30 min, 1 h, 2 h, 3 h, and 4 h in animals after dosing on day 0 and thereafter once daily for clinical signs and twice daily for mortality for 14 days study period. The body weights of all animals were recorded on day 0 (prior to dosing) and on days 7 and 14. At the end of the experimental period, all the animals were euthanized by an overdose of carbon dioxide (CO_2_) and subjected to gross pathology examination, for external and internal observation [22].

### 2.5. Subacute or 28 days Repeated Dose Toxicity Study

The subacute oral toxicity study was conducted as per the OECD guidelines 407 [23]. Based on the dose range finding study, the main study doses were selected: low dose (125 mg/kg b.w), mid dose (250 mg/kg b.w), and high dose (500 mg/kg b.w). In the main study, sixty Wistar rats (30 males and 30 females), were distributed into six groups (5 animals/sex/group). The animals were 7 to 8 weeks old, weighing between 150 and 185 g (males) and 137–168 g (females) at the start of the study. The grouping and respective doses are explained in Table 1 and their body weight was recorded.

Dose administration was done once daily for a period of four weeks through oral gavage using an intubation cannula. Corn oil was used as a vehicle. Control animals were administered with the vehicle alone. The formulation of AC^3^® was prepared fresh daily, and the vehicle was administered at the dose volume of 10 mL/kg body weight. There was no administration of AC^3^® formulation or vehicle during the 14 days recovery period. During the study period, all animals were examined twice daily for morbidity and daily for clinical changes if any. Body weight and feed consumption of animals were recorded. Blood samples taken in dipotassium ethylenediaminetetraacetic acid (K2 EDTA) vacutainer centrifuge tubes were used for hematological examination. Blood samples taken in lithium-heparinized centrifuge tubes were centrifuged; plasma was separated and used for biochemical analysis. Urine analysis was also conducted. All animals were weighed and sacrificed by exsanguinations under CO_2_, and sent to necropsy. The findings from detailed necropsy of all group animals were recorded. Tissues and organs collected were preserved in 10% neutral buffered formalin solution, testes were fixed in modified Davidson's fixative, and eyes were fixed in Davidson's fixative on completion of the gross pathology examination from all animals.

### 2.6. Subchronic or 90 days Repeated Toxicity Study

The subchronic toxicity study was conducted as per the OECD guidelines 408 [24]. In this study, 120 Wistar rats (60 males and 60 females) of 7-8 weeks old, weighing between 130–170 g (males) and 130–175 g (females), were distributed into six groups (10 animals/sex/group). The dose grouping of the animals is shown in Table 2.

The AC^3^® was administered by oral gavage once daily for a period of 90 consecutive days. Corn oil was used as a vehicle. Control animals were administered with the vehicle alone. Recovery group animals were observed for a further 28 days after completion of the treatment to check for any reversibility of treatment-related change/delayed test item related toxicity. There was no administration of AC^3^® formulation/vehicle during the 14 days recovery period. During the study period, all animals were examined twice daily for mortality and once daily for clinical signs of toxicity. They were subjected to detailed clinical observation once before the start of treatment and weekly thereafter during the treatment and recovery period. Neurological and ophthalmological examinations were also conducted. Body weight and feed consumption were recorded weekly. Analysis of blood (hematology and clinical biochemistry) and urine was performed at the end of three months and during the recovery period. All animals were sacrificed by exsanguinations under CO_2_, necropsy was carried out, and the weight of various organs was recorded. Tissues and organs collected on completion of the gross pathology examination from all animals were preserved in a 10% buffered neutral formalin solution. Testes were fixed in modified Davidson's fixative, and eyes were fixed in Davidson's fixative. Histopathological examination was performed on the preserved organs and tissues of all animals in the control and high-dose groups. Since there were no treatment-related changes in the high-dose group, examinations were not extended to animals of other dose groups and recovery groups also. All gross lesions were examined and recorded.

### 2.7. Reproductive/Developmental Toxicity Study

The reproductive/developmental toxicity study was conducted as per the OECD guidelines 421 [25]. Eighty Wistar rats (40 males and 40 females (nulliparous and nonpregnant with uniform estrous cyclicity)) were distributed into four groups (10 animals/sex/group) as mentioned in Table 3.

During the study period, the animals weighed 230–293 g (males, 13 weeks old) and 197–269 g (females, 16 weeks old), respectively. Control animals were administered with the vehicle (corn oil) alone. The animals were administered with vehicle and AC^3^® by oral route for a period of 28 consecutive days (males) and ≈63 days (females) during premating, mating, gestation, and up to day 13 postpartum. The dose volume was maintained at 10 mL/kg body weight in all the groups. During the experimental period, all animals were checked weekly for body weight and food consumption during gestation and lactation. Clinical signs were observed daily, and mortality was observed twice daily. The litters were examined thoroughly after delivery for gross abnormalities in live births, stillbirths, runts, and the sex of live pups. The duration of gestation was recorded. Live pups were counted and sexed, and litters were weighed on days 0, 4, and 13 postpartum. The anogenital distance (AGD) of each pup was measured on the postnatal day 4, and the number of nipples/areolae in male pups was counted and recorded on postnatal day 13. In addition, any abnormal behaviors of the pups were also recorded. All animals were weighed and sacrificed by exsanguinations under CO_2_, and necropsy was carried out. A detailed histological examination was performed on the ovaries, uterus with cervix, testes, and epididymidis of the animals of control and high-dose group. The other preserved organs including the thyroid from pups were also examined.

### 2.8. Bacterial Reverse Mutation Assay

To evaluate the mutagenicity of AC^3^®, a bacterial reverse mutation assay in two independent tests was performed as per the OECD guidelines 471 [26], with five bacterial test strains (*Salmonella typhimurium*: TA98, TA100, TA1535, TA1537, and TA102) using the plate-incorporating method. Trial I was conducted both in the presence (5% v/v cofactor supplemented postmitochondrial S9 fraction) and absence of metabolic activation, and Trial II was conducted only in the presence (10% v/v cofactor supplemented postmitochondrial S9 fraction) of metabolic activation. The preliminary cytotoxicity assay was used to establish the dose range for the mutagenicity assay, based on which the mutagenicity assay (Trial I and II) was conducted. Based on the solubility test, dimethyl sulfoxide was selected as the vehicle for the test item. In both Trial I and Trial II, the following five concentrations of the test item were tested in solvent vehicle control (dimethyl sulfoxide), negative control (distilled water), and concurrent positive controls in triplicates: 0.002, 0.005, 0.010, 0.020, and 0.039 mg/plate.

### 2.9. Mammalian Bone Marrow Micronucleus Test

The mammalian bone marrow micronucleus test was performed as per the OECD guidelines 474 [27]. After one-week acclimatization of Swiss albino mice (body weight: 23–39 g & age: 7–10 weeks), a dose range-finding experiment was conducted using four groups (3 animals/sex/group). Based on the results of the dose range study, the main experiment was conducted using five groups (5 animals/sex/group). The vehicle control group (corn oil) and treatment groups were treated at the dose levels of 500 mg (low), 1000 mg (mid), and 2000 mg (high) of AC^3^®/kg body weight, respectively, via oral route twice at an approximately 24 hours interval. The positive control group (cyclophosphamide monohydrate-50 mg/kg body weight) was treated once on day 2 via the intraperitoneal route. The animals were sacrificed by CO_2_ asphyxiation, and samples of bone marrow were collected from the femurs. A needle with a syringe filled with fetal bovine serum was inserted through the opening at the lower end of the femur bone to rinse the marrow into the prelabeled tubes. The cells were centrifuged at 1500 rpm for 10 min, and the supernatant was discarded. A few drops of residual supernatant were used to resuspend the bone marrow cells. Two bone marrow slides were prepared for each animal, air dried for 15–20 min, fixed in absolute methanol for 10 min, and stained with 5% Giemsa. For each animal, the proportion of immature erythrocytes among total erythrocytes was determined by counting a total of at least 500 erythrocytes. Clinical observation, mortality, body weight, P/E ratio, and micronucleus induction were evaluated.

## 3. Statistical Analysis

The data have been subjected to statistical analysis for the significance of test item-induced changes and their interpretation of potential for toxicity. Statistical analysis of the experimental data was performed in GraphPad Prism version 8.0.1 (244). The data for each group of animals was subjected to analysis of variance (ANOVA), followed by Dunnett's test and an unpaired *t*-test as a post hoc comparison. All the values are expressed as mean ± standard deviation (SD). Statistical comparisons were evaluated at the 5% (*P* ≤ 0.05) significance level.

## 4. Results

### 4.1. Acute Toxicity

In an acute toxicity study, oral administration at a dose of 2000 mg/kg body weight resulted in no mortality and did not exhibit any clinical signs of toxicity in female Wistar rats. There were no abnormalities found in the organs during necropsy. During the study period, body weight gain was observed on day 7 and day 14 as compared to day 0 as shown in Table 4.

### 4.2. Subacute Toxicity

**Figure 3:**
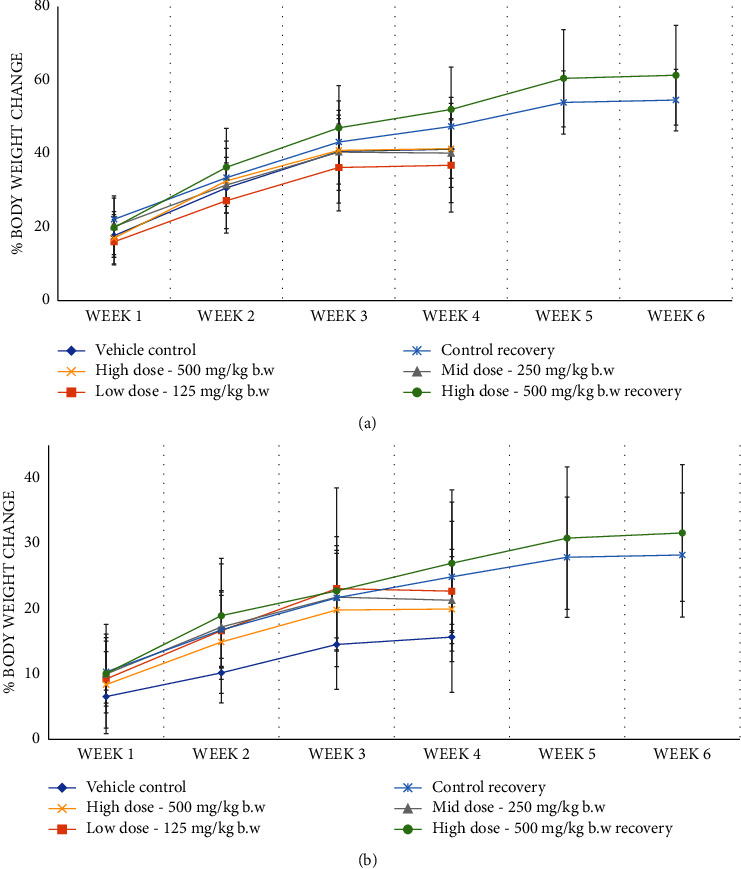
(a) Effect of AC^3^®on the percentage of body weight change of male rats in subacute toxicity. Values are expressed as mean ± SD, *n* = 5. (b) Effect of AC^3^® on the percentage of body weight change of female rats in subacute toxicity. Values are expressed as mean ± SD, *n* = 5.

In a subacute or repeated dose 28-day toxicity study, clinical signs of toxicity and mortality were not observed up to 500 mg/kg in any of the treatment or control group animals. During the study and recovery period, there was no significant change in the body weight, body weight gain percentage (Figures 3(a) and 3(b)), and feed consumption between the treated and control groups.

During the study, we observed minor changes in the hematological parameters (Table 5); however, they were not considered treatment-related as these were neither consistent nor dose-dependent. Also, no treatment-related changes were noticed in biochemical (Table 6), urine analysis, in absolute (Table 7), and relative organ weights of internal organs at necropsy. Gross and histopathological examination also showed no significant lesion.

### 4.3. Subchronic Toxicity

**Figure 4:**
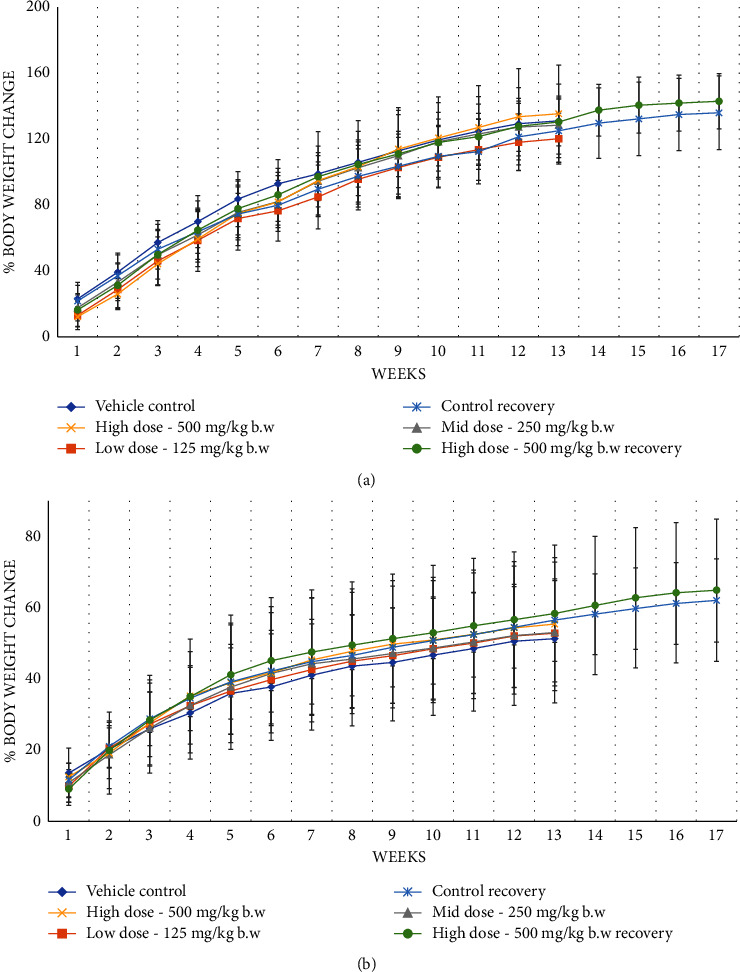
(a) Effect of AC^3^® on the percentage of body weight change of male rats in subchronic toxicity. Values are expressed as mean ± SD, *n* = 10. (b) Effect of AC^3^® on the percentage of body weight change of female rats in subchronic toxicity. Values are expressed as mean ± SD, *n* = 10.

In a subchronic or 90-day repeated dose toxicity study, oral administration showed no mortality and clinical signs of toxicity. No significant difference was observed in feed consumption (Supplementary Table S1), body weight, as well as percentage body weight change (Figures 4(a) and 4(b)).

Similarly, there were no significant changes in neurological, ophthalmological, hematological (Table 8), and biochemical parameters (Table 9) as well as urine analysis.

The necropsy did not reveal any treatment-related pathological significance. There were no treatment-related changes observed in absolute (Table 10) and relative organ weights.

### 4.4. Reproductive/Developmental Toxicity Study

In the reproductive/developmental toxicity study, no treatment-related clinical signs of toxicity and mortality were observed in any of the groups. In females, no significant changes were observed in body weight during gestation and lactation period (Table 11).

Also, there were no changes in the absolute organ weights of males (testes and epididymidis) and females (ovaries and uterus) (Table 12) between the control and treated groups. The reproductive/developmental parameters of the animals like the mean number of implantations, number of pregnancies, number of dams littered, gestation length, and number of days of pregnancy were comparable as shown in Table 13.

### 4.5. Bacterial Reverse Mutation Assay

In the reverse mutation assay, both the experimental methods I and II did not significantly increase the mean number of revertant colony counts of bacterial test strains neither in the presence (5–10% v/v S9) nor absence of the metabolic activation. Furthermore, no trend of an increased number of revertant colonies with increased dosing (0.002, 0.005, 0.010, 0.020, and 0.039 mg/plate) of the test item was observed (Supplementary Tables: S2a and S2b). These results indicated that the AC^3^® did not induce mutagenicity in any of the test strains and thus is nonmutagenic.

### 4.6. Mammalian Bone Marrow Micronucleus Test

In the mammalian bone marrow micronucleus study, all treated (500, 1000, and 2000 mg of AC^3^®/kg) animals were observed to be normal throughout the experimental period. Bone marrow toxicity was not observed in any of the treatment groups. There was no statistically significant increase in the number of micronucleated polychromatic erythrocytes in either male or female animals compared to vehicle control (Supplementary Tables: S3a and S3b).

## 5. Discussion

Turmeric (*Curcuma longa*), the golden colored indispensable ingredient, is one of the most consumed spices in India and Asia and has been used as traditional medicine over centuries. The research found that curcumin is typically accompanied by DMC and BDMC―the two other curcumin analogs present in turmeric that provide health benefits. Several studies on turmeric extracts and curcumin have been investigated for their toxicity and were found to be safe [28–30]. In the present study, AC^3^® is a mixture of phytonutrients that expounds the benefits of BDMC, as the major ingredient, along with DMC and curcumin. Compared to curcumin and DMC, BDMC has better stability in the physiological medium [31] and is found to be more bioavailable [32]. Although many efficacy studies have been conducted on BDMC, there were no published toxicity profiles for this molecule. So, the current study was proposed to study the preclinical safety profile of AC^3^®.

In a single-dose acute toxicity study, results indicated that AC^3^® was nontoxic up to 2000 mg/kg body weight. The clinical signs of toxicity and mortality, feed consumption, body weight gain, and gross pathology were found to be normal during the study period in the female adult rats. Further observations for 14 days also revealed no toxic effects. Similarly, in a 28-day, subacute study, AC^3^® at doses of up to 500 mg/kg caused no mortality and abnormal clinical signs in animals. No significant change was observed in body weight gain and feed consumption during both the treatment and recovery periods. During the study, no treatment-related changes were observed in hematological and biochemical parameters except for some changes in a few animals which cannot be attributed to AC^3^®. In histopathological examination, few animals revealed minimal mononuclear cell infiltration, perivascular and minimal alveolar histiocytosis in the lungs, minimal tubular regeneration and/or degeneration in the cortex, and minimal inflammation in the urothelium part of the kidneys, as well as minimal myocardial degeneration in the heart. However, these changes were not considered treatment-related as these changes were neither consistent nor dose-dependent. There were no treatment-related changes observed in organ weight (absolute and relative) and urine analysis when compared with the respective control group animals.

In a 90-day, subchronic toxicity study, AC^3^® at doses of up to 500 mg/kg body weight caused no mortality and clinical signs of abnormalities in animals. The changes in body weight and feed consumption were comparable among the control and treatment groups. Changes in hematological parameters were observed in a few animals like a decrease in lymphocytes and eosinophils and an increase in neutrophils. Similarly, few animals showed a decrease (in alanine aminotransferase, aspartate aminotransferase, and total bilirubin) and an increase (in calcium, thyroid stimulating hormone (TSH), and blood urea nitrogen) in clinical biochemistry parameters. The organ weight (absolute and relative) of the animals was comparable among the treated and control groups, except for a few animals that showed a decrease in adrenals (relative organ weight) and an increase in pituitary gland (absolute organ weight). Histopathological examination in a few animals showed minimal mononuclear cell infiltration and mixed cell infiltration with early alveolar fibrosis (lungs) and minimal inflammation of the urothelium (kidneys). All these changes were not considered treatment-related as these changes were neither consistent nor dose-dependent. The functional observational battery (FOB) parameters and urine analysis did not reveal any treatment-related changes in comparison with the respective control groups Thus, the results obtained showed that AC^3^® can be characterized as safe in the 90-day subchronic study with no observed adverse effect level (NOAEL) of 500 mg/kg body weight.

In a reproductive/developmental toxicity study, AC^3^® at doses of up to 500 mg/kg body weight showed no clinical signs of toxicity or mortality during the study. The body weight, percentage body weight change, and food consumption in animals in the treatment groups were comparable to those in the control group. The organ weight as well as the histopathological changes in reproductive organs of the high dose was comparable to those of the control group. The reproductive parameters like mating performance, gestation length, mean number of corpora lutea, mean implantation, mean litter size, mean litter/pup weight, AGD, nipples/areolae in male pups, implantation losses, and the sex ratio of offspring in treatment groups were also comparable to control. Also, the thyroxine (T4) and TSH levels did not reveal any significant changes. The gross external examination of the pups sacrificed on day 4 and day 13 postpartum did not reveal any abnormalities. External and internal examinations (male and female animals) of the control and treatment groups did not reveal any lesions of pathological significance. From the results obtained, AC^3^® was found to be safe with no observed adverse effect in the reproductive or developmental stages of the Wistar rats.

In the bacterial reverse mutation study, AC^3^® is nonmutagenic, as it does not induce (point) gene mutations by base-pair changes or frameshift in the histidine operon in any of the five test strains of *Salmonella typhimurium* (either with or without metabolic activation). Likewise, in a mammalian bone marrow micronucleus study, AC^3^® did not induce micronuclei formation either in male or female animals treated with dose levels up to 2000 mg/kg body weight, justifying it as nonmutagenic for clastogenicity and aneugenicity.

## 6. Conclusion

The findings from the acute study revealed that oral doses up to 2000 mg/kg body weight of AC^3^® showed no adverse effect or treatment-related mortality in animals. As per the Globally Harmonized System of Classification and Labelling of Chemicals (GHS), the test item can be classified as GHS category 5 or unclassified. AC^3^® in the repeated dose (28 and 90 days) and in the reproductive/developmental toxicity study showed no signs of toxicity. In these studies, the “No Observed Adverse Effect Level” (NOAEL) was considered up to 500 mg/kg body weight in both male and female animals. In addition, AC^3^® was found to be a nonmutagenic based on mammalian bone marrow micronucleus test and a bacterial reverse mutation assay.

## Figures and Tables

**Table 1:** The table represents the grouping of the doses and number of animals used for subacute toxicity study of AC^3^®.

Group no.	Treatment groups	Dose (mg/kg b.w)	Sex	No. of rats
G1	Vehicle control	0	M	5
			F	5

G2	Low dose	125	M	5
			F	5

G3	Mid dose	250	M	5
			F	5

G4	High dose	500	M	5
			F	5

G1R^*∗*^	Vehicle control	0	M	5
			F	5

G4R^*∗*^	High dose	500	M	5
			F	5

M: male; F: female; ^*∗*^recovery groups with the 14 days recovery period.

**Table 2:** The table represents the grouping of the doses and number of animals used for subchronic toxicity study of AC^3^®.

Group no.	Treatment groups	Dose (mg/kg b.w)	Sex	No. of rats
G1	Vehicle control	0	M	10
			F	10

G2	Low dose	125	M	10
			F	10

G3	Mid dose	250	M	10
			F	10

G4	High dose	500	M	10
			F	10

G1R^*∗*^	Vehicle control	0	M	10
			F	10

G4R^*∗*^	High dose	500	M	10
			F	10

M: male; F: female; ^*∗*^recovery groups with the 14 days recovery period.

**Table 3:** The table represents the graded doses administered and number of animals used for reproductive/developmental toxicity study of AC^3^®.

Group no.	Treatment groups	Dose (mg/kg b.w)	Sex	No. of rats
G1	Vehicle control	0	M	10
			F	10

G2	Low dose	125	M	10
			F	10

G3	Mid dose	250	M	10
			F	10

G4	High dose	500	M	10
			F	10

M: male; F: female.

**Table 4:** Effect of single dose oral gavage to AC^3^® on body weight and body weight change (%) in female Wistar rats.

Study type	Animal no.	Dose (mg/kg b.w)	Body weight (g) on day			Body weight change (%) on day	
			0	7	14	0 to 7	0 to 14
Step 1	1	2000	187.35	203.22	219.68	8.47	17.26
	2		179.47	195.15	208.23	8.74	16.02
	3		174.71	190.69	208.97	9.15	19.61

Mean ± SD			**180.51** **±** **6.38**	**196.35** **±** **6.35**	**212.29** **±** **6.41**	**8.78** **±** **0.34**	**17.63** **±** **1.82**

Step 2	4	2000	195.48	211.63	232.36	8.26	18.87
	5		183.20	199.87	214.87	9.10	17.29
	6		188.92	206.98	221.95	9.27	17.48

Mean ± SD			**189.20** **±** **6.14**	**205.98** **±** **5.89**	**223.06** **±** **8.80**	**8.88** **±** **0.54**	**17.88** **±** **0.86**

Values are expressed as mean ± SD. Bold values highlight the mean values of the three readings of individual rodent.

**Table 5:** Effect of 28 days exposure to AC3® on hematological parameters in Wistar rats.

Parameter	Sex	Vehicle control	Treated			Control recovery	High dose-500 mg/kg b.w recovery
			Low dose-125 mg/kg b.w	Mid dose-250 mg/kg b.w	High dose-500 mg/kg b.w		
WBC (10^9^/L)	M	11.93 ± 1.92	12.94 ± 1.09	14.12 ± 1.77	16.90 ± 3.23^*∗*^	11.59 ± 5.17	13.17 ± 4.72
	F	14.29 ± 2.65	11.51 ± 3.83	13.23 ± 4.27	13.93 ± 2.88	14.93 ± 2.76	12.49 ± 4.43

RBC (10^12^/L)	M	8.45 ± 0.75	8.25 ± 0.34	8.47 ± 0.43	8.50 ± 0.54	9.04 ± 0.26	8.85 ± 0.63
	F	8.01 ± 0.54	8.31 ± 0.55	8.23 ± 0.37	8.12 ± 0.29	8.66 ± 0.47	8.57 ± 0.22

Hemoglobin (g/L)	M	148.00 ± 14.40	145.20 ± 4.55	145.80 ± 4.64	146.80 ± 2.95	158.00 ± 5.07	155.40 ± 8.20
	F	144.40 ± 4.83	147.40 ± 6.58	147.40 ± 5.22	144.20 ± 3.70	151.80 ± 4.60	151.20 ± 4.71

Haematocrit (L/L)	M	0.47 ± 0.04	0.47 ± 0.01	0.46 ± 0.03	0.47 ± 0.02	0.494 ± 0.02	0.485 ± 0.04
	F	0.44 ± 0.03	0.46 ± 0.03	0.46 ± 0.02	0.46 ± 0.01	0.464 ± 0.01	0.467 ± 0.02

MCV (fL)	M	55.84 ± 1.11	56.76 ± 2.06	54.74 ± 0.04	55.74 ± 1.93	54.62 ± 0.96	54.82 ± 1.00
	F	55.40 ± 1.86	55.82 ± 1.57	55.80 ± 0.71	56.42 ± 1.16	53.68 ± 1.76	54.42 ± 1.51

MCH (g/L)	M	17.50 ± 0.43	17.62 ± 0.54	17.22 ± 0.45	17.30 ± 0.87	17.50 ± 0.47	17.58 ± 0.38
	F	18.08 ± 1.06	17.76 ± 0.81	17.92 ± 0.42	17.80 ± 0.43	17.52 ± 0.64	17.68 ± 0.38

MCHC (g/L)	M	313.40 ± 4.88	310.60 ± 7.60	314.20 ± 6.14	310.80 ± 6.72	320.20 ± 7.26	320.80 ± 7.73
	F	326.00 ± 12.75	318.20 ± 8.17	321.00 ± 7.78	315.60 ± 4.04	326.40 ± 3.97	324.40 ± 5.68

Platelet count (10^9^/L)	M	862.80 ± 176.20	891.60 ± 149.13	997.40 ± 203.50	959.20 ± 156.55	1030.0 ± 190.90	1133.80 ± 92.82
	F	966.60 ± 63.69	979.20 ± 118.27	1030.80 ± 41.59	984.40 ± 155.09	1084.0 ± 149.83	1041.60 ± 216.59

Neutrophils (%)	M	13.38 ± 5.83	18.74 ± 2.42	14.76 ± 5.63	25.40 ± 30.15^*∗*^	15.34 ± 7.09	17.16 ± 5.71
	F	14.14 ± 4.08	16.24 ± 7.65	11.96 ± 3.05	13.38 ± 2.58	11.78 ± 2.29	20.08 ± 5.15^**≠**^

Lymphocytes (%)	M	82.50 ± 6.74	87.28 ± 2.62	81.14 ± 6.42	68.96 ± 28.98	79.74 ± 7.39	78.46 ± 5.96
	F	81.72 ± 4.05	78.70 ± 9.39	82.60 ± 3.77	81.66 ± 2.51	83.64 ± 2.67	72.90 ± 4.93^**≠**^

Monocytes (%)	M	1.84 ± 0.67	1.22 ± 0.22	1.48 ± 0.69	1.62 ± 0.41	1.86 ± 0.59	1.80 ± 0.57
	F	1.68 ± 0.34	1.64 ± 0.86	1.68 ± 0.68	1.96 ± 0.40	1.82 ± 0.36	2.18 ± 0.63

Eosinophils (%)	M	0.62 ± 0.36	0.48 ± 0.40	0.44 ± 0.23	0.20 ± 0.12	0.98 ± 0.47	0.74 ± 0.09
	F	0.52 ± 0.28	1.16 ± 0.97	0.80 ± 0.40	0.70 ± 0.45	0.58 ± 0.40	1.70 ± 1.43

Leucocytes (%)	M	1.54 ± 0.34	2.22 ± 0.63	2.04 ± 0.23	3.68 ± 2.42^*∗*^	1.98 ± 0.49	1.76 ± 0.45
	F	1.84 ± 0.54	2.16 ± 0.58	2.82 ± 1.07	2.26 ± 0.84	2.06 ± 0.72	3.06 ± 0.92

Basophils (%)	M	0.08 ± 0.04	0.04 ± 0.05	0.08 ± 0.04	0.08 ± 0.04	0.10 ± 0.00	0.08 ± 0.04
	F	0.10 ± 0.00	0.08 ± 0.04	0.06 ± 0.05	0.12 ± 0.04	0.10 ± 0.00	0.08 ± 0.04

Reticulocyte count (%)	M	2.29 ± 0.49	2.52 ± 0.40	2.03 ± 0.81	1.83 ± 0.35	1.86 ± 0.47	2.06 ± 0.58
	F	2.49 ± 0.82	2.10 ± 0.59	2.07 ± 0.18	2.14 ± 0.29	1.89 ± 0.52	1.95 ± 0.44

Prothrombin time (sec)	M	19.88 ± 1.57	18.70 ± 3.94	20.86 ± 1.11	20.10 ± 2.14	18.22 ± 1.58	18.02 ± 1.63
	F	20.34 ± 0.83	20.28 ± 1.66	20.18 ± 1.21	19.40 ± 0.99	18.40 ± 1.36	16.98 ± 0.72

Clotting time (sec)	M	144.00 ± 25.10	144.00 ± 25.10	144.00 ± 25.10	150.00 ± 30.00	144.00 ± 25.10	144.00 ± 25.10
	F	150.00 ± 30.00	144.00 ± 39.12	144.00 ± 25.10	144.00 ± 25.10	144.00 ± 25.10	138.00 ± 26.83

WBC: total leukocyte count; MCV: mean corpuscular volume; MCH: mean corpuscular hemoglobin; MCHC: mean corpuscular hemoglobin concentration; RBC: erythrocyte count. Values are expressed as mean ± SD, *n* = 5. M: male; F: female. ^*∗*^Significant from vehicle control (*P* ≤ 0.05) in male; ^≠^Significant from vehicle control recovery (*P* ≤ 0.05) in female.

**Table 6:** Effect of 28 days exposure to AC^3^® on biochemical parameters in Wistar rats.

Parameter	Sex	Vehicle control	Treated			Control recovery	High dose-500 mg/kg b.w recovery
			Low dose-125 mg/kg b.w	Mid dose-250 mg/kg b.w	High dose-500 mg/kg b.w		
Albumin (g/L)	M	29.20 ± 2.12	28.86 ± 1.37	29.64 ± 0.65	29.94 ± 1.24	29.88 ± 1.38	29.02 ± 1.58
	F	30.40 ± 1.39	30.80 ± 1.37	30.28 ± 1.38	29.74 ± 1.53	29.38 ± 1.32	30.86 ± 0.88

Alanine amino transferase (U/L)	M	47.80 ± 5.22	43.00 ± 6.28	52.80 ± 9.26	44.20 ± 1.92	39.80 ± 5.72	47.40 ± 6.19
	F	46.40 ± 6.69	45.20 ± 10.35	39.40 ± 6.15	44.80 ± 5.36	42.20 ± 5.12	45.60 ± 12.28

Aspartate amino transferase (U/L)	M	103.40 ± 10.81	100.20 ± 13.74	151.20 ± 28.80^*∗*^	104.60 ± 11.24	110.40 ± 20.11	94.00 ± 30.66
	F	125.20 ± 20.78	103.20 ± 15.21	101.40 ± 6.95	106.40 ± 22.80	99.00 ± 31.29	122.40 ± 24.52

Calcium (mmol/L)	M	2.46 ± 0.07	2.45 ± 0.07	2.51 ± 0.05	2.48 ± 0.04	2.52 ± 0.05	2.54 ± 0.07
	F	2.50 ± 0.09	2.49 ± 0.05	2.49 ± 0.03	2.47 ± 0.08	2.50 ± 0.10	2.52 ± 0.05

Total cholesterol (mmol/L)	M	1.39 ± 0.11	1.54 ± 0.20	1.51 ± 0.13	1.70 ± 0.15^*∗*^	1.50 ± 0.23	1.84 ± 0.21
	F	1.30 ± 0.27	1.33 ± 0.16	1.40 ± 0.25	1.46 ± 0.14	1.84 ± 0.26	1.76 ± 0.52

Creatinine kinase (U/L)	M	177.60 ± 77.29	372.20 ± 139.83	607.80 ± 617.04	460.80 ± 239.19	193.80 ± 166.58	248.60 ± 193.76
	F	305.80 ± 194.60	231.20 ± 121.59	236.20 ± 53.83	275.60 ± 108.39	237.60 ± 230.61	348.00 ± 255.53

Creatinine (*µ*mol/L)	M	24.40 ± 2.30	22.60 ± 1.82	24.20 ± 3.27	25.20 ± 3.35	28.00 ± 4.00	27.80 ± 2.68
	F	30.60 ± 9.71	26.20 ± 4.32	27.40 ± 3.91	30.40 ± 7.47	30.40 ± 2.19	32.20 ± 4.55

Gamma-glutamyl transferase (U/L)	M	1.80 ± 0.45	1.60 ± 0.55	1.60 ± 0.55	1.60 ± 0.55	1.20 ± 0.45	1.00 ± 0.00
	F	1.60 ± 0.89	1.40 ± 0.55	1.40 ± 0.55	1.20 ± 0.84	1.00 ± 0.71	0.40 ± 0.55

Glucose (mmol/L)	M	6.79 ± 0.72	6.99 ± 1.20	7.56 ± 0.54	7.90 ± 0.49	8.26 ± 2.10	7.47 ± 1.33
	F	6.91 ± 1.15	6.72 ± 0.78	6.81 ± 1.02	7.05 ± 0.86	7.00 ± 0.90	6.87 ± 1.43

Phosphorus (mmol/L)	M	1.82 ± 0.16	1.75 ± 0.23	1.69 ± 0.09	1.80 ± 0.13	2.03 ± 0.40	1.82 ± 0.07
	F	1.39 ± 0.19	1.41 ± 0.18	1.37 ± 0.11	1.44 ± 0.11	1.71 ± 0.20	1.57 ± 0.28

Lactate dehydrogenase (U/L)	M	84.60 ± 18.66	127.00 ± 53.94	146.60 ± 80.66	111.60 ± 37.59	116.60 ± 59.54	89.80 ± 31.79
	F	144.8 ± 40.24	86.00 ± 24.85	78.60 ± 17.53	124.60 ± 66.43	120.20 ± 66.39	154.20 ± 89.75

Total bilirubin (*µ*mol/L)	M	2.77 ± 0.14	2.52 ± 0.15	2.81 ± 0.28	2.78 ± 0.21	2.46 ± 0.44	2.79 ± 0.50
	F	3.26 ± 0.46	2.97 ± 0.16	2.92 ± 0.31	2.72 ± 0.46	2.74 ± 0.49	3.01 ± 0.69

Total protein (g/L)	M	67.90 ± 3.28	65.86 ± 2.04	71.72 ± 4.15	65.98 ± 2.22	66.10 ± 3.71	66.24 ± 3.12
	F	70.74 ± 3.55	69.00 ± 2.26	68.30 ± 1.08	66.34 ± 2.84	65.44 ± 3.74	68.68 ± 3.92

Triglycerides (mmol/L)	M	0.83 ± 0.20	0.96 ± 0.18	0.78 ± 0.27	0.68 ± 0.14	0.50 ± 0.17	1.25 ± 0.42^*∗*^
	F	0.44 ± 0.14	0.48 ± 0.21	0.50 ± 0.24	0.64 ± 0.23^**≠**^	0.88 ± 0.47	0.79 ± 0.31

Blood urea nitrogen (mmol/L)	M	4.76 ± 0.97	4.97 ± 1.42	4.86 ± 0.54	5.63 ± 1.38	6.64 ± 1.64	6.37 ± 0.95
	F	4.60 ± 1.59	4.65 ± 0.57	5.85 ± 1.56	5.86 ± 0.86	7.25 ± 1.09	7.16 ± 1.33

Sodium (mmol/L)	M	140.42 ± 1.34	139.86 ± 1.01	141.08 ± 1.79	138.64 ± 1.43	141.90 ± 1.92	144.00 ± 2.92
	F	138.72 ± 1.29	139.02 ± 1.36	138.76 ± 0.94	139.52 ± 1.72	140.58 ± 3.00	141.40 ± 3.93

Potassium (mmol/L)	M	3.61 ± 0.34	3.93 ± 0.85	3.49 ± 0.20	3.87 ± 0.22	4.14 ± 0.66	3.97 ± 0.34
	F	3.75 ± 0.42	3.40 ± 0.12	3.53 ± 0.40	3.45 ± 0.22	3.91 ± 0.40	3.96 ± 0.61

Values are expressed as mean ± SD, *n* = 5. M: male; F: female. ^*∗*^Significant from vehicle control and control recovery (*P* ≤ 0.05) in male; ^≠^Significant from vehicle control (*P* ≤ 0.05) in female.

**Table 7:** Effect of 28 days exposure to AC^3^® on absolute organ weights (g) in Wistar rats.

Organs	Sex	Vehicle control	Treated			Control recovery	High dose-500 mg/kg b.w recovery
			Low dose-125 mg/kg b.w	Mid dose-250 mg/kg b.w	High dose-500 mg/kg b.w		
Adrenals	M	0.061 ± 0.015	0.069 ± 0.014	0.069 ± 0.008	0.074 ± 0.010	0.055 ± 0.014	0.045 ± 0.007
	F	0.074 ± 0.018	0.069 ± 0.020	0.083 ± 0.022	0.056 ± 0.011	193.69 ± 9.60	200.47 ± 28.37

Liver	M	8.716 ± 1.269	8.904 ± 1.523	8.907 ± 1.657	8.875 ± 1.499	8.250 ± 0.954	8.318 ± 1.010
	F	6.114 ± 0.690	6.481 ± 0.749	6.773 ± 0.197	6.315 ± 0.638	0.504 ± 0.222	0.423 ± 0.044

Kidneys	M	1.773 ± 0.173	1.818 ± 0.422	1.705 ± 0.283	1.632 ± 0.341	1.646 ± 0.175	1.737 ± 0.067
	F	1.214 ± 0.133	1.287 ± 0.194	1.313 ± 0.169	1.242 ± 0.176	5.63 ± 0.77	6.11 ± 0.87

Heart	M	0.878 ± 0.018	0.878 ± 0.175	1.022 ± 0.197	0.896 ± 0.259	0.846 ± 0.060	0.977 ± 0.124
	F	0.610 ± 0.094	0.750 ± 0.126	0.734 ± 0.114	0.676 ± 0.145	1.175 ± 0.135	1.211 ± 0.151

Brain	M	1.859 ± 0.062	1.849 ± 0.144	1.720 ± 0.090	1.732 ± 0.163	1.726 ± 0.076	1.791 ± 0.118
	F	1.715 ± 0.053	1.688 ± 0.138	1.744 ± 0.083	1.705 ± 0.124	0.70 ± 0.11	0.67 ± 0.10

Spleen	M	0.711 ± 0.174	0.741 ± 0.104	0.894 ± 0.299	0.673 ± 0.146	0.748 ± 0.383	0.723 ± 0.185
	F	0.620 ± 0.127	0.582 ± 0.190	0.626 ± 0.154	0.490 ± 0.134	1.702 ± 0.068	1.732 ± 0.055

Thymus	M	0.366 ± 0.081	0.305 ± 0.120	0.347 ± 0.078	0.390 ± 0.113	0.370 ± 0.094	0.319 ± 0.047
	F	0.267 ± 0.051	0.364 ± 0.058	0.375 ± 0.100	0.309 ± 0.040	0.56 ± 0.12	0.50 ± 0.10

Ovaries	F	0.117 ± 0.007	0.122 ± 0.023	0.133 ± 0.012	0.140 ± 0.012	0.062 ± 0.024	0.060 ± 0.014

Uterus with cervix	F	0.558 ± 0.185	0.499 ± 0.023	0.133 ± 0.012	0.140 ± 0.012	0.10 ± 0.05	0.12 ± 0.04

Prostate	M	0.488 ± 0.133	0.500 ± 0.070	0.538 ± 0.192	0.465 ± 0.091	0.549 ± 0.132	0.490 ± 0.161

Seminal vesicle with coagulation gland	M	0.536 ± 0.170	0.584 ± 0.144	0.639 ± 0.255	0.489 ± 0.184	0.667 ± 0.194	0.650 ± 0.149

Testes	M	3.167 ± 0.201	3.046 ± 0.264	3.107 ± 0.227	2.775 ± 0.524	2.984 ± 0.203	3.034 ± 0.269

Epididymis	M	1.026 ± 0.051	1.135 ± 0.093	1.124 ± 0.144	0.900 ± 0.239	1.063 ± 0.117	0.987 ± 0.168

Values are expressed as mean ± SD, *n* = 5. M: male; F: female.

**Table 8:** Effect of 90 days exposure to AC^3^® on hematological parameters in Wistar rats.

Parameter	Sex	Vehicle control	Treated			Control recovery	High dose-500 mg/kg b.w recovery
			Low dose-125 mg/kg b.w	Mid dose-250 mg/kg b.w	High dose-500 mg/kg b.w		
WBC (10^9^/L)	M	12.47 ± 2.53	11.92 ± 3.14	10.94 ± 2.87	10.55 ± 3.99	11.00 ± 2.74	9.82 ± 3.50
	F	7.94 ± 2.63	8.38 ± 2.12	8.25 ± 3.27	8.39 ± 2.58	8.92 ± 1.84	9.44 ± 2.64

RBC (10^12^/L)	M	9.15 ± 0.51	9.35 ± 0.46	9.10 ± 0.49	9.09 ± 0.28	9.63 ± 0.48	9.65 ± 0.48
	F	8.09 ± 0.56	8.31 ± 0.38	8.37 ± 0.37	8.52 ± 0.46	8.56 ± 0.40	8.62 ± 0.33

Hemoglobin (g/L)	M	150.90 ± 3.87	154.10 ± 5.80	149.00 ± 6.88	152.20 ± 3.91	160.90 ± 7.06	157.10 ± 2.02
	F	138.80 ± 10.90	140.80 ± 5.59	143.10 ± 4.33	145.10 ± 7.36	146.60 ± 5.85	147.70 ± 4.22

Haematocrit (L/L)	M	0.483 ± 0.02	0.500 ± 0.02	0.484 ± 0.02	0.489 ± 0.01	0.52 ± 0.02	0.51 ± 0.01
	F	0.447 ± 0.03	0.452 ± 0.02	0.460 ± 0.02	0.465 ± 0.02	0.47 ± 0.02	0.48 ± 0.02

MCV (fL)	M	52.79 ± 1.57	53.48 ± 2.15	53.28 ± 1.61	53.83 ± 1.44	54.13 ± 1.81	53.10 ± 1.67
	F	55.21 ± 1.76	54.52 ± 1.47	55.04 ± 1.40	54.62 ± 1.87	55.26 ± 1.32	55.20 ± 1.31

MCH (g/L)	M	16.52 ± 0.92	16.49 ± 0.83	16.40 ± 0.58	16.73 ± 0.58	16.73 ± 0.69	16.31 ± 0.74
	F	17.16 ± 0.55	17.00 ± 0.56	17.09 ± 0.43	17.05 ± 0.67	17.12 ± 0.46	17.13 ± 0.47

MCHC (g/L)	M	313.00 ± 10.62	308.30 ± 5.33	307.60 ± 4.65	310.60 ± 6.64	308.80 ± 6.18	306.80 ± 5.20
	F	310.80 ± 4.37	311.80 ± 5.65	310.80 ± 3.79	311.90 ± 5.43	309.90 ± 3.78	310.20 ± 6.55

Platelet count (10^9^/L)	M	928.40 ± 94.46	943.90 ± 231.45	1012.30 ± 121.84	1034.90 ± 120.88	948.70 ± 195.72	1053.60 ± 140.93
	F	968.90 ± 273.27	1018.80 ± 118.33	1095.50 ± 134.80	1095.70 ± 142.01	991.20 ± 163.15	1089.90 ± 114.91

Neutrophils (%)	M	16.75 ± 3.21	17.81 ± 4.38	20.49 ± 5.23	18.27 ± 4.27	17.40 ± 3.10	21.69 ± 4.19^*∗*^
	F	17.30 ± 4.12	17.55 ± 3.80	20.37 ± 5.86	20.50 ± 8.59	18.76 ± 6.46	21.38 ± 5.79

Lymphocytes (%)	M	78.49 ± 4.49	78.35 ± 5.55	75.71 ± 5.49	78.00 ± 4.47	76.39 ± 3.63	70.92 ± 3.67^*∗*^
	F	76.46 ± 5.12	76.78 ± 5.51	74.83 ± 6.24	73.79 ± 9.02	73.78 ± 7.51	68.30 ± 4.82

Monocytes (%)	M	1.87 ± 0.85	1.92 ± 0.84	1.90 ± 0.71	1.65 ± 0.51	3.08 ± 1.64	2.46 ± 0.89
	F	2.03 ± 0.57	1.61 ± 0.37	1.87 ± 0.69	2.05 ± 0.71	3.34 ± 1.32	3.23 ± 0.79

Eosinophils (%)	M	1.85 ± 1.67	0.86 ± 0.47	0.92 ± 0.31	0.98 ± 0.77	1.74 ± 0.99	3.64 ± 2.70
	F	2.55 ± 2.35	3.00 ± 3.35	1.98 ± 1.44	2.65 ± 2.35	2.38 ± 1.20	5.40 ± 3.43^**≠**^

Leukocyte count (%)	M	0.97 ± 0.16	1.02 ± 0.27	0.96 ± 0.23	1.11 ± 0.47	1.32 ± 0.43	1.23 ± 0.34
	F	1.62 ± 2.05	1.01 ± 0.41	0.88 ± 0.35	0.95 ± 0.44	1.68 ± 0.71	1.67 ± 0.59

Basophils (%)	M	0.05 ± 0.05	0.07 ± 0.05	0.02 ± 0.04	0.03 ± 0.05	0.05 ± 0.05	0.06 ± 0.05
	F	0.04 ± 0.05	0.05 ± 0.05	0.03 ± 0.05	0.03 ± 0.05	0.05 ± 0.05	0.04 ± 0.05

Reticulocyte count (%)	M	1.69 ± 0.47	1.86 ± 0.63	2.00 ± 0.49	1.74 ± 0.27	1.52 ± 0.35	1.29 ± 0.43
	F	2.17 ± 1.58	1.71 ± 0.67	1.82 ± 0.69	1.45 ± 0.32	1.57 ± 0.51	1.56 ± 0.51

Clotting time (sec)	M	147.00 ± 29.83	153.00 ± 22.14	144.00 ± 23.66	147.00 ± 26.27	153.00 ± 22.14	150.00 ± 20.00
	F	150.00 ± 20.00	147.00 ± 22.14	147.00 ± 22.14	144.00 ± 23.66	147.00 ± 22.14	147.00 ± 22.14

WBC: total leukocyte count; MCV: mean corpuscular volume; MCH: mean corpuscular hemoglobin; MCHC: mean corpuscular hemoglobin concentration; RBC: erythrocyte count. Values are expressed as mean ± SD, *n* = 10; M: male; F: female. ^*∗*^^,≠^Significant from control recovery (*P* ≤ 0.05) in male and female.

**Table 9:** Effect of 90 days exposure to AC^3^® on biochemical parameters in Wistar rats.

Parameter	Sex	Vehicle control	Treated			Control recovery	High dose-500 mg/kg b.w recovery
			Low dose-125 mg/kg b.w	Mid dose-250 mg/kg b.w	High dose-500 mg/kg b.w		
Albumin (g/L)	M	29.45 ± 1.81	31.32 ± 1.97	30.82 ± 2.56	30.66 ± 1.51	30.13 ± 3.11	28.67 ± 2.71
	F	33.66 ± 3.08	34.63 ± 2.62	33.46 ± 3.72	34.80 ± 2.52	30.90 ± 4.39	31.39 ± 3.75

Alanine amino transferase (U/L)	M	68.30 ± 16.97	50.20 ± 12.12^*∗*^	51.00 ± 10.51^*∗*^	48.40 ± 9.67^*∗*^	52.90 ± 14.19	52.20 ± 13.55
	F	55.60 ± 19.86	63.80 ± 19.46	51.20 ± 9.54	42.80 ± 6.89	47.90 ± 8.69	38.80 ± 13.05

Aspartate amino transferase (U/L)	M	127.00 ± 21.26	103.40 ± 15.69^*∗*^	105.70 ± 13.52^*∗*^	105.30 ± 13.41^*∗*^	100.20 ± 17.00	102.70 ± 11.61
	F	128.70 ± 70.05	113.40 ± 25.48	121.10 ± 44.47	105.90 ± 20.47	118.90 ± 21.24	95.00 ± 26.89^**≠**^

Calcium (mmol/L)	M	2.47 ± 0.07	2.55 ± 0.04^*∗*^	2.48 ± 0.09	2.50 ± 0.05	2.34 ± 0.08	2.31 ± 0.07
	F	2.54 ± 0.07	2.56 ± 0.09	2.56 ± 0.06	2.56 ± 0.06	2.33 ± 0.09	2.36 ± 0.09

Total cholesterol (mmol/L)	M	1.68 ± 0.29	1.73 ± 0.35	1.65 ± 0.27	1.65 ± 0.26	1.51 ± 0.37	1.46 ± 0.40
	F	1.98 ± 0.36	2.04 ± 0.33	1.90 ± 0.46	1.92 ± 0.39	1.65 ± 0.45	1.50 ± 0.23

Creatinine kinase (U/L)	M	118.60 ± 27.45	176.70 ± 108.05	104.40 ± 25.36	170.90 ± 145.91	218.50 ± 116.89	149.60 ± 73.45
	F	223.60 ± 237.12	88.70 ± 24.05	94.80 ± 30.12	119.40 ± 61.63	148.10 ± 74.44	161.10 ± 128.21

Creatinine (*µ*mol/L)	M	27.90 ± 3.48	26.10 ± 3.60	24.30 ± 2.45	27.30 ± 4.27	25.60 ± 2.17	26.00 ± 3.40
	F	29.50 ± 3.75	26.80 ± 3.55	26.40 ± 2.27	28.10 ± 3.03	25.90 ± 3.84	29.30 ± 3.80

Gamma-glutamyl transferase (U/L)	M	0.30 ± 0.48	0.30 ± 0.48	0.20 ± 0.42	0.10 ± 0.32	0.50 ± 0.53	0.80 ± 0.42
	F	0.50 ± 0.97	0.10 ± 0.32	0.30 ± 0.48	0.50 ± 0.71	0.80 ± 0.42	0.70 ± 0.48

Glucose (mmol/L)	M	7.11 ± 0.45	7.05 ± 0.81	7.64 ± 0.64	8.08 ± 0.91^*∗*^	5.97 ± 0.56	6.16 ± 0.84
	F	7.03 ± 0.89	7.50 ± 0.86	7.52 ± 1.08	7.39 ± 0.89	5.98 ± 0.95	5.96 ± 0.76

High-density lipoprotein (mg/dL)	M	0.95 ± 0.16	1.02 ± 0.21	0.98 ± 0.19	1.01 ± 0.19	0.92 ± 0.20	0.87 ± 0.24
	F	1.27 ± 0.27	1.32 ± 0.21	1.23 ± 0.31	1.24 ± 0.24	1.04 ± 0.31	0.99 ± 0.13

Phosphorus (mmol/L)	M	1.90 ± 0.14	1.83 ± 0.15	1.97 ± 0.29	1.88 ± 0.17	1.64 ± 0.13	1.56 ± 0.17
	F	1.52 ± 0.26	1.40 ± 0.14	1.46 ± 0.22	1.50 ± 0.25	1.38 ± 0.13	1.34 ± 0.19

Lactate dehydrogenase (U/L)	M	122.20 ± 22.19	131.40 ± 78.26	104.90 ± 30.76	93.90 ± 34.89	90.90 ± 25.76	72.40 ± 11.58
	F	154.40 ± 58.18	101.40 ± 32.88	146.90 ± 76.63	109.50 ± 48.68	85.20 ± 19.62	98.30 ± 46.71

Low-density lipoprotein (mg/dL)	M	0.44 ± 0.09	0.41 ± 0.10	0.39 ± 0.10	0.35 ± 0.05	0.39 ± 0.13	0.41 ± 0.11
	F	0.36 ± 0.06	0.37 ± 0.08	0.33 ± 0.09	0.33 ± 0.08	0.38 ± 0.11	0.29 ± 0.09

Total bilirubin (*µ*mol/L)	M	2.62 ± 0.33	2.65 ± 0.61	2.59 ± 0.22	2.64 ± 0.21	2.85 ± 0.29	2.69 ± 0.27
	F	3.12 ± 0.52	2.88 ± 0.30	2.69 ± 0.35^**≠**^	2.76 ± 0.18	2.94 ± 0.50	2.66 ± 0.42

Total protein (g/L)	M	70.70 ± 2.64	70.57 ± 2.48	69.39 ± 3.07	70.03 ± 2.72	69.40 ± 3.33	68.25 ± 2.66
	F	74.92 ± 4.62	76.06 ± 3.75	74.93 ± 4.24	74.49 ± 1.70	73.33 ± 3.94	71.18 ± 3.18

Triglycerides (mmol/L)	M	0.50 ± 0.11	0.52 ± 0.23	0.47 ± 0.15	0.48 ± 0.26	0.46 ± 0.15	0.38 ± 0.11
	F	0.44 ± 0.10	0.41 ± 0.22	0.37 ± 0.16	0.36 ± 0.07	0.36 ± 0.12	0.45 ± 0.18

Blood urea nitrogen (mmol/L)	M	6.57 ± 1.11	6.22 ± 1.46	7.12 ± 1.30	7.16 ± 1.07	5.12 ± 0.87	5.12 ± 1.31
	F	7.52 ± 1.38	6.95 ± 1.40	7.33 ± 1.81	7.67 ± 0.86	5.37 ± 1.39	6.69 ± 1.05^**≠**^

Sodium (mmol/L)	M	141.13 ± 1.42	140.83 ± 1.85	140.73 ± 1.65	141.59 ± 1.98	142.77 ± 0.87	142.99 ± 1.28
	F	140.55 ± 1.45	140.53 ± 1.14	140.56 ± 1.54	140.50 ± 2.20	143.15 ± 1.04	142.78 ± 0.69

Potassium (mmol/L)	M	3.76 ± 0.26	3.83 ± 0.37	3.79 ± 0.22	3.78 ± 0.24	3.55 ± 0.12	3.74 ± 0.26
	F	3.46 ± 0.30	3.41 ± 0.20	3.75 ± 0.34	3.40 ± 0.20	3.46 ± 0.15	3.61 ± 0.28

T3 (triiodothyronine) (ng/dL)	M	44.03 ± 0.62	43.81 ± 0.35	43.94 ± 0.36	44.68 ± 0.32	43.99 ± 0.53	43.95 ± 0.35
	F	43.96 ± 0.56	44.01 ± 0.41	43.75 ± 0.24	44.05 ± 0.26	43.79 ± 0.38	43.77 ± 0.26

T4 (thyroxine) (ng/dL)	M	6759.20 ± 7.87	6760.82 ± 6.75	6761.78 ± 9.89	6764.65 ± 6.47	6760.50 ± 3.75	6763.82 ± 7.63
	F	6765.28 ± 5.36	6760.87 ± 5.90	6766.36 ± 8.23	6762.27 ± 8.82	6765.25 ± 9.43	6761.47 ± 5.84

TSH (thyroid stimulating hormone) (ng/mL)	M	1.73 ± 0.71	1.57 ± 0.43	1.54 ± 0.64	1.79 ± 0.68	2.72 ± 0.63	2.21 ± 1.32
	F	1.58 ± 0.61	2.34 ± 0.47^**≠**^	2.15 ± 0.70	1.57 ± 0.77	1.39 ± 0.54	2.20 ± 1.15

Values are expressed as mean ± SD, *n* = 10; M: male; F: female.^*∗*^^,≠^Significant from vehicle control (*P* ≤ 0.05) in male and female. ^**≠**^Significant from control recovery (*P* ≤ 0.05) in female.

**Table 10:** Effect of 90 days exposure to AC^3^® on absolute organ weights (g) in Wistar rats.

Organs	Sex	Vehicle control	Treated			Control recovery	High dose-500 mg/kg b.w recovery
			Low dose-125 mg/kg b.w	Mid dose-250 mg/kg b.w	High dose-500 mg/kg b.w		
Adrenals	M	0.060 ± 0.010	0.065 ± 0.010	0.069 ± 0.015	0.061 ± 0.011	0.055 ± 0.009	0.052 ± 0.007
	F	0.070 ± 0.010	0.073 ± 0.009	0.069 ± 0.016	0.058 ± 0.011	0.058 ± 0.006	0.059 ± 0.005

Liver	M	9.580 ± 1.070	9.473 ± 1.689	10.285 ± 1.192	9.717 ± 2.196	8.599 ± 1.286	8.812 ± 1.289
	F	6.765 ± 0.622	6.518 ± 0.689	6.510 ± 0.325	6.365 ± 0.870	6.060 ± 0.852	5.868 ± 1.015

Kidneys	M	1.970 ± 0.250	1.925 ± 0.288	2.111 ± 0.250	2.167 ± 0.303	2.022 ± 0.199	1.981 ± 0.351
	F	1.483 ± 0.213	1.320 ± 0.141	1.380 ± 0.216	1.443 ± 0.155	1.413 ± 0.225	1.337 ± 0.238

Heart	M	1.120 ± 0.120	1.056 ± 0.129	1.190 ± 0.188	1.141 ± 0.141	1.094 ± 0.246	1.088 ± 0.134
	F	0.725 ± 0.095	0.744 ± 0.096	0.775 ± 0.088	0.776 ± 0.074	0.749 ± 0.081	0.769 ± 0.125

Brain	M	1.830 ± 0.080	1.833 ± 0.162	1.893 ± 0.111	1.890 ± 0.089	1.860 ± 0.096	1.891 ± 0.103
	F	1.750 ± 0.081	1.742 ± 0.102	1.824 ± 0.158	1.797 ± 0.143	1.784 ± 0.071	1.808 ± 0.134

Spleen	M	0.830 ± 0.130	0.759 ± 0.227	0.771 ± 0.173	0.812 ± 0.202	0.702 ± 0.135	0.737 ± 0.097
	F	0.591 ± 0.283	0.607 ± 0.125	0.514 ± 0.127	0.526 ± 0.006	0.609 ± 0.171	0.536 ± 0.092

Thymus	M	0.290 ± 0.060	0.341 ± 0.077	0.300 ± 0.069	0.342 ± 0.112	0.276 ± 0.041	0.280 ± 0.090
	F	0.300 ± 0.086	0.296 ± 0.088	0.268 ± 0.100	0.276 ± 0.004	0.259 ± 0.087	0.261 ± 0.059

Thyroid parathyroid gland	M	0.020 ± 0.000	0.024 ± 0.005	0.026 ± 0.005	0.026 ± 0.007	0.022 ± 0.003	0.025 ± 0.004
	F	0.022 ± 0.006	0.024 ± 0.003	0.024 ± 0.005	0.023 ± 0.004	0.023 ± 0.002	0.022 ± 0.004

Pituitary gland	M	0.010 ± 0.000	0.011 ± 0.001	0.011 ± 0.001	0.011 ± 0.001	0.010 ± 0.001	0.013 ± 0.001^*∗*^
	F	0.013 ± 0.003	0.012 ± 0.002	0.013 ± 0.002	0.014 ± 0.011	0.011 ± 0.002	0.012 ± 0.002

Ovaries	F	0.174 ± 0.137	0.115 ± 0.023	0.242 ± 0.314	0.133 ± 0.030	0.110 ± 0.017	0.112 ± 0.012

Uterus with cervix	F	0.852 ± 0.348	0.813 ± 0.464	0.738 ± 0.325	0.736 ± 0.323	0.713 ± 0.178	0.621 ± 0.172

Testes	M	2.850 ± 0.430	3.059 ± 0.358	2.968 ± 0.297	3.138 ± 0.317	2.943 ± 0.258	3.115 ± 0.323

Prostate	M	0.660 ± 0.260	0.666 ± 0.224	0.776 ± 0.293	0.836 ± 0.397	0.827 ± 0.330	0.800 ± 0.248

Seminal vesicle with coagulation gland	M	1.050 ± 0.400	0.827 ± 0.248	0.960 ± 0.240	0.934 ± 0.293	0.914 ± 0.193	0.868 ± 0.212

Epididymis	M	1.390 ± 0.300	1.226 ± 0.241	1.231 ± 0.204	1.283 ± 0.188	1.279 ± 0.115	1.350 ± 0.255

Values are expressed as mean ± SD, *n* = 10. M: male; F: female. ^*∗*^Significant from control recovery (*P* ≤ 0.05) in male.

**Table 11:** Effect of body weight (g) of female animals during gestation and lactation period in reproductive/developmental toxicity study of AC^3^®.

Treatment groups	Gestation period (days)				Lactation period (days)		
	0	7	14	20	0	4	13
Vehicle control	244.07 ± 12.19	250.06 ± 12.32	272.82 ± 15.08	299.06 ± 22.90	221.78 ± 22.09	227.05 ± 21.84	243.97 ± 23.63
Low dose-125 mg/kg b.w	237.42 ± 9.76	243.84 ± 9.66	268.59 ± 7.59	302.36 ± 17.92	225.83 ± 17.45	232.56 ± 18.99	254.52 ± 16.73
Mid dose-250 mg/kg b.w	241.84 ± 10.94	252.63 ± 11.23	265.36 ± 10.77	295.95 ± 10.76	227.99 ± 14.15	235.28 ± 15.25	246.58 ± 14.18
High dose-500 mg/kg b.w	235.73 ± 9.77	246.09 ± 10.88	270.77 ± 17.64	300.61 ± 21.30	221.60 ± 15.49	230.61 ± 18.97	250.82 ± 18.18

Values are expressed as mean ± SD. *n* = 10 for vehicle control, low, and high dose; *n* = 9 for mid dose.

**Table 12:** Effect of absolute organ weights (g) of animals in reproductive/developmental toxicity study of AC^3^®.

Treatment groups	Male			Female	
	Testes	Epididymidis	Seminal vesicle & coagulation gland	Ovaries	Uterus with cervix
Vehicle control	2.997 ± 0.309	1.247 ± 0.097	1.948 ± 0.448	0.098 ± 0.014	0.471 ± 0.220
Low dose-125 mg/kg b.w	2.866 ± 0.403	1.175 ± 0.199	1.817 ± 0.492	0.103 ± 0.014	0.557 ± 0.225
Mid dose-250 mg/kg b.w	2.878 ± 0.477	1.143 ± 0.183	1.989 ± 0.383	0.085 ± 0.019	0.436 ± 0.113
High dose-500 mg/kg b.w	3.012 ± 0.371	1.278 ± 0.252	1.968 ± 0.346	0.088 ± 0.017	0.499 ± 0.410

Values are expressed as mean ± SD for the treatment groups. *n* = 10 (for both male and female animals).

**Table 13:** Effect of AC^3^® on different parameters of reproductive/developmental toxicity study.

Observations		Values			
Dosage (units)		Vehicle control	Low dose-125 mg/kg b.w	Mid dose-250 mg/kg b.w	High dose-500 mg/kg b.w
Pairs started (N)		10	10	10	10
Females showing evidence of copulation (N)		10	10	10	10
Females achieving pregnancy (N)		10	10	09	10
Conceiving days 1–5 (N)		3	4	3	6
Conceiving days ≥6 (N)		7	6	6	4
Pregnancy ≤21 Days (N)		3	2	2	2
Pregnancy = 22 Days (N)		6	7	6	7
Pregnancy ≥23 Days (N)		1	1	1	1
Dams with live young born (N)		10	10	9	10
Dams with live young at day 4 PP (N)		10	10	9	10
Implants/dam (mean)		11.80	12.50	11.22	12.60
Live pups/dam at birth (mean)		11.20	11.50	10.56	11.50
Live pups/dam at day 4 (mean)		11.10	11.50	10.44	11.50
Sex ratio (M/F) at birth (mean)		105.19	110.05	104.07	106.92
Sex ratio (M/F) at day 4 (mean)		102.69	110.05	102.08	106.92
Litter weight at birth (mean)		69.90	72.83	63.61	75.07
Litter weight at day 4 (mean)		84.23	95.07	78.63	98.12
Pup weight at birth (mean)		6.24	6.33	6.03	6.53
Pup weight at the time of AGD measurement (mean) male/females	M	7.61	8.17	7.69	8.48
	F	7.55	8.37	7.57	8.59
Pup AGD on the same postnatal day, birth–day 4 (mean male/females note postnatal day)	M	2.84	2.85	2.88	2.83
	F	1.82	1.84	1.86	1.83
Pup weight at day 4 (mean)		7.59	8.27	7.58	8.53
Male pup nipple retention at day 13 (mean)		0	0	0	0
Pup weight at day 13 (mean)		18.72	19.09	19.73	19.91
Abnormal pups					
Dams with 0 (N)		10	10	9	10
Dams with 1 (N)		0	0	0	0
Dams with ≥2 (N)		0	0	0	0
Loss of offspring					
Prenatal/postimplantations (implantations minus live births)					
Females with 0		6	3	7	7
Females with 1		3	4	1	1
Females with 2		0	3	1	2
Females with ≥3		1	0	1	0
Postnatal (live births minus alive at postnatal day 13)					
Females with 0		9	10	9	10
Females with 1		1	0	1	0
Females with 2		0	0	0	0
Females with ≥3		0	0	0	0

M: male; F: female; N: number; PP: postpartum; AGD: anogenital distance.

## Data Availability

The data used to support the findings of this study are included within the article.

## Supplementary Materials

Supplementary Materials Table S1: effect of 90 days exposure to AC^3^® on weekly feed consumption (g/r/d) in Wistar rats. Table S2a: effect of AC^3^® on mean revertant colony count in trial I (Plate Incorporation Method). Table S2b: effect of AC^3^® on mean revertant colony count in trial II (Plate Incorporation Method). Table S3a: effect of AC^3^® on bone marrow micronucleus test in Swiss Albino male mice. Table S3b: effect of AC^3^® on bone marrow micronucleus test in Swiss Albino female mice.
