# Supplementary material for: Assessment of Safety Profile of Activated Curcumin C3 Complex (AC3®), Enriched Extract of Bisdemethoxycurcumin from the Rhizomes of Curcuma longa
Source: J Toxicol. 2023 Oct 31;2023:3729399. doi: 10.1155/2023/3729399 (PMC10629997; doi:10.1155/2023/3729399)
Supplement: Supplementary Materials — Table S1: effect of 90 days exposure to AC3® on weekly feed consumption (g/r/d) in Wistar rats. Table S2a: effect of AC3® on mean revertant colony count in trial I (Plate Incorporation Method). Table S2b: effect of AC3® on mean revertant colony count in trial II (Plate Incorporation Method). Table S3a: effect of AC3® on bone marrow micronucleus test in Swiss Albino male mice. Table S3b: effect of AC3® on bone marrow micronucleus test in Swiss Albino female mice. [file 3729399.f1.docx]

**Supplementary Tables**

**Table S1: Effect of 90 days exposure to AC^3®^ on weekly feed consumption (g/r/d) in Wistar rats.**

| **Week** | **Sex** | **Vehicle**  **control** | **Treated** | | | **Control recovery** | **High dose -**  **500 mg/kg b.w recovery** |
| --- | --- | --- | --- | --- | --- | --- | --- |
|  |  |  | **Low dose -**  **125 mg/kg b.w** | **Mid dose -**  **250 mg/kg b.w** | **High dose -**  **500 mg/kg b.w** |  |  |
| **1** | M | 16.20±1.37 | 14.88±2.07 | 14.41±2.35 | 13.03±1.50 | 15.38±2.56 | 13.58±1.99 |
|  | F | 14.01±1.15 | 11.89±2.62 | 11.22±2.07 | 12.46±1.62 | 14.53±0.29 | 13.60±0.97 |
| **2** | M | 17.28±1.18 | 16.25±1.32 | 15.64±2.37 | 14.69±1.34 | 17.74±1.89 | 14.93*±0.95 |
|  | F | 14.49±1.28 | 13.87±2.34 | 11.90±1.57 | 14.24±2.64 | 15.05±0.13 | 13.82±1.09 |
| **3** | M | 16.29±1.55 | 14.92±0.75 | 16.43±3.21 | 17.20±1.39 | 15.33±2.55 | 17.71±1.55 |
|  | F | 14.29±1.67 | 14.62±0.52 | 14.96±1.60 | 13.11±1.47 | 14.94±0.62 | 14.02±1.63 |
| **4** | M | 19.17±1.19 | 17.52±0.82 | 18.75±1.94 | 18.56±1.34 | 17.95±1.46 | 19.27±0.96 |
|  | F | 15.06±1.82 | 14.97±0.54 | 15.30±1.28 | 14.08±1.89 | 15.67±1.57 | 15.39±2.29 |
| **5** | M | 17.22±2.10 | 16.21±1.22 | 16.17±1.90 | 18.04±2.65 | 15.78±2.30 | 16.87±1.36 |
|  | F | 14.27±1.80 | 12.54±1.34 | 13.30±0.74 | 12.37±0.84 | 13.86±1.03 | 13.15±2.55 |
| **6** | M | 17.71±1.98 | 16.24±0.55 | 17.36±2.02 | 17.81±1.31 | 15.40±1.86 | 17.19±2.26 |
|  | F | 12.48±1.37 | 11.84±1.09 | 13.54±1.11 | 13.47±1.30 | 12.99±0.14 | 12.84±2.41 |
| **7** | M | 17.80±1.22 | 18.06±1.22 | 19.05±1.92 | 19.07±1.90 | 17.41±1.30 | 18.28±1.67 |
|  | F | 13.33±1.59 | 12.57±1.36 | 14.48±1.39 | 13.57±1.02 | 13.68±1.06 | 13.44±1.46 |
| **8** | M | 18.28±1.35 | 18.75±1.49 | 17.67±2.11 | 17.58±2.80 | 17.37±2.16 | 17.48±1.41 |
|  | F | 13.22±2.22 | 12.61±1.80 | 12.37±1.03 | 12.47±0.68 | 13.38±1.50 | 12.70±2.02 |
| **9** | M | 18.22±0.92 | 17.79±1.50 | 17.50±2.84 | 19.36±2.46 | 16.93±1.56 | 17.83±1.79 |
|  | F | 13.96±1.61 | 12.17±1.36 | 12.62±1.49 | 12.08±1.20 | 13.67±2.26 | 13.50±1.45 |
| **10** | M | 18.00±1.16 | 17.27±1.58 | 16.34±2.65 | 18.12±2.95 | 17.69±3.36 | 17.23±1.70 |
|  | F | 13.72±1.73 | 11.77±1.00 | 12.76±1.90 | 11.98±0.72 | 13.48±1.96 | 12.92±1.16 |
| **11** | M | 17.62±0.51 | 16.94±1.23 | 16.47±1.48 | 19.78±1.81 | 16.47±1.33 | 18.59±1.96 |
|  | F | 13.48±1.18 | 11.56±0.85 | 12.57±1.58 | 12.92±0.96 | 13.65±1.65 | 13.41±1.31 |
| **12** | M | 20.60±3.63 | 17.04±1.68 | 16.75±1.70 | 20.06±2.86 | 19.89±2.47 | 17.96±3.10 |
|  | F | 13.87±0.93 | 12.11±1.32 | 12.34±3.63 | 13.28±0.79 | 14.26±2.20 | 13.53±1.41 |
| **13** | M | 23.25± 1.17 | 23.56 ±1.55 | 21.37±2.71 | 22.61±3.12 | 20.50±1.92 | 19.25±1.39 |
|  | F | 17.75±1.13 | 16.40±1.32 | 16.48±0.97 | 15.78±0.71 | 15.65±1.82 | 14.55±1.30 |
| **14** | M | - | - | - | - | 20.76±2.13 | 20.18±1.07 |
|  | F | - | - | - | - | 17.15±2.61 | 15.40±2.95 |
| **15** | M | - | - | - | - | 20.97±1.83 | 20.71±1.44 |
|  | F | - | - | - | - | 18.90±2.91 | 18.11±1.32 |
| **16** | M | - | - | - | - | 20.50±1.85 | 20.97±1.01 |
|  | F | - | - | - | - | 16.74±1.96 | 16.72±1.33 |
| **17** | M | - | - | - | - | 19.89±0.51 | 19.32±1.46 |
|  | F | - | - | - | - | 15.30±2.10 | 14.19±1.68 |

Values are expressed as mean ± SD, n=10. M: Male; F: Female. *Significant from control recovery (P≤ 0.05) in male.

**Table S2a: Effect of AC^3®^ on mean revertant colony count in trial I (Plate Incorporation Method).**

| **Test Item**  **Concentration**  **(mg/plate)** | **Absence of metabolic activation (-S9)** | | | | | **Presence of metabolic activation (+S9 10% v/v S9 Mix)** | | | | |
| --- | --- | --- | --- | --- | --- | --- | --- | --- | --- | --- |
|  | **TA 98** | **TA 100** | **TA 1535** | **TA 1537** | **TA 102** | **TA 98** | **TA 100** | **TA 1535** | **TA 1537** | **TA 102** |
|  | **Mean ± SD** | **Mean ± SD** | **Mean ± SD** | **Mean ± SD** | **Mean±SD** | **Mean±SD** | **Mean±SD** | **Mean±SD** | **Mean±SD** | **Mean±SD** |
| **Negative Control**  **(Distilled water)** | 21±3.06 | 101±6.66 | 13±0.58 | 7±1.15 | 237±8.72 | 21±2.89 | 98±7.51 | 13±0.58 | 6±1.00 | 235±5.29 |
| **Vehicle Control**  **(Dimethyl sulfoxide)** | 21±2.08 | 101±4.73 | 13±1.53 | 6±1.15 | 241±2.52 | 21±2.65 | 96±3.06 | 13±0.58 | 7±1.15 | 234±6.56 |
| **T1 (0.01953125)** | 20±3.61 | 100±3.79 | 12±1.15 | 7±1.15 | 237±8.50 | 19±1.15 | 94±3.06 | 12±1.15 | 6±1.15 | 234±4.93 |
| **T2 (0.0390625)** | 19±1.15 | 102±4.51 | 13±1.53 | 6±1.15 | 230±6.51 | 20±3.00 | 95±7.09 | 13±1.53 | 6±0.00 | 230±4.58 |
| **T3 (0.078125)** | 20±3.06 | 98±4.04 | 11±1.73 | 6±0.58 | 228±3.61 | 20±1.00 | 93±3.51 | 12±1.73 | 7±1.15 | 232±7.00 |
| **T4 (0.15625)** | 21±3.06 | 95±6.03 | 12±1.00 | 5±1.73 | 218±10.54 | 19±2.65 | 91±2.52 | 12±0.58 | 5±1.00 | 231±6.51 |
| **T5 (0.3125)** | 15±1.53 | 87±7.02 | 10±0.58 | 9±11.27 | 195±8.62 | 16±0.58 | 77±3.00 | 9±0.58 | 2±1.15 | 194±10.97 |
| **Positive Control** | 316±9.61 | 676±9.61 | 314±12.22 | 195±8.62 | 1636±20.52 | 316±6.43 | 675±15.70 | 304±7.00 | 198±8.33 | 1632±38.89 |

T1-T5 = Test item concentration from lower to higher.

**Positive Controls:** Absence of metabolic activation :2-Nitrofluorene (TA98); Sodium azide (TA100 & TA1535); 9-Aminoacridine (TA1537); Mitomycin-C (TA102); Presence of metabolic activation (Benzo[a]pyrene- TA98, TA100, TA1535, TA1537 and TA102).

**Table S2b: Effect of AC^3®^ on mean revertant colony count in trial II (Plate Incorporation Method).**

| **Test Item**  **Concentration**  **(mg/plate)** | **Presence of metabolic activation (+S9 10% v/v S9 Mix)** | | | | |
| --- | --- | --- | --- | --- | --- |
|  | **TA 98** | **TA 100** | **TA 1535** | **TA 1537** | **TA 102** |
|  | **Mean±SD** | **Mean**±**SD** | **Mean**±**SD** | **Mean**±**SD** | **Mean**±**SD** |
| **Negative Control**  **(Distilled water)** | 21±3.00 | 99±6.66 | 13±2.00 | 7±1.15 | 225±8.62 |
| **Vehicle Control (Dimethyl sulfoxide)** | 20±3.00 | 97±5.51 | 13±1.15 | 7±0.58 | 232±3.61 |
| **T1 (0.01953125)** | 19±0.58 | 102±7.81 | 12±1.15 | 6±1.73 | 231±6.03 |
| **T2 (0.0390625)** | 19±3.46 | 93±4.36 | 13±0.58 | 7±1.15 | 230±4.04 |
| **T3 (0.078125)** | 19±2.31 | 92±5.57 | 12±1.53 | 7±0.00 | 229±13.58 |
| **T4 (0.15625)** | 19±2.52 | 92±3.21 | 12±1.73 | 6±1.53 | 212±9.02 |
| **T5 (0.3125)** | 15±0.58 | 84±3.46 | 9±1.15 | 3±0.58 | 191±5.13 |
| **Positive Control** | 314±6.24 | 723±6.51 | 317±10.02 | 201±13.61 | 1709±31.75 |

T1-T5 = Test item concentration from lower to higher. **Positive Control:** Benzo[a]pyrene - TA98, TA100, TA1535, TA1537 and TA102 (presence of metabolic activation).

**Table S3a: Effect of AC^3®^ on bone marrow micronucleus test in Swiss Albino male mice.**

| **Dose**  **(mg/kg b.w)** | **Animal No.** | **PCE** | **NCE** | **P/E** | **MNPCE** | **Total PCE** | **%** **MNPCE** | **No. of MNPCE/ Group** | **MNPCE** |
| --- | --- | --- | --- | --- | --- | --- | --- | --- | --- |
|  |  |  |  |  |  |  |  |  | **Mean ± SD** |
| **0**  **(Vehicle Control)** | 25 | 268 | 245 | 0.522 | 1 | 4000 | 0.03 | 1 | 0.2±0.45 |
|  | 26 | 262 | 248 | 0.514 | 0 | 4000 | 0.00 |  |  |
|  | 27 | 268 | 238 | 0.530 | 0 | 4000 | 0.00 |  |  |
|  | 28 | 255 | 246 | 0.509 | 0 | 4000 | 0.00 |  |  |
|  | 29 | 264 | 238 | 0.526 | 0 | 4000 | 0.00 |  |  |
| **500** | 30 | 262 | 246 | 0.516 | 1 | 4000 | 0.03 | 2 | 0.4±0.55 |
|  | 31 | 258 | 250 | 0.508 | 0 | 4000 | 0.00 |  |  |
|  | 32 | 255 | 248 | 0.507 | 0 | 4000 | 0.00 |  |  |
|  | 33 | 263 | 243 | 0.520 | 1 | 4000 | 0.03 |  |  |
|  | 34 | 260 | 244 | 0.516 | 0 | 4000 | 0.00 |  |  |
| **1000** | 35 | 245 | 257 | 0.488 | 0 | 4000 | 0.00 | 2 | 0.4±0.89 |
|  | 36 | 248 | 253 | 0.495 | 0 | 4000 | 0.00 |  |  |
|  | 37 | 263 | 248 | 0.515 | 0 | 4000 | 0.00 |  |  |
|  | 38 | 248 | 256 | 0.492 | 0 | 4000 | 0.00 |  |  |
|  | 39 | 261 | 248 | 0.513 | 2 | 4000 | 0.05 |  |  |
| **2000** | 40 | 258 | 252 | 0.506 | 1 | 4000 | 0.03 | 3 | 0.6±0.89 |
|  | 41 | 248 | 253 | 0.495 | 0 | 4000 | 0.00 |  |  |
|  | 42 | 260 | 243 | 0.517 | 2 | 4000 | 0.05 |  |  |
|  | 43 | 266 | 238 | 0.528 | 0 | 4000 | 0.00 |  |  |
|  | 44 | 237 | 265 | 0.472 | 0 | 4000 | 0.00 |  |  |
| **50**  **(Positive Control – Cyclophosphamide monohydrate)** | 45 | 248 | 256 | 0.492 | 27 | 4000 | 0.68 | 134 | 26.8±1.10 |
|  | 46 | 253 | 254 | 0.499 | 27 | 4000 | 0.68 |  |  |
|  | 47 | 244 | 260 | 0.484 | 27 | 4000 | 0.68 |  |  |
|  | 48 | 256 | 254 | 0.502 | 25 | 4000 | 0.63 |  |  |
|  | 49 | 261 | 248 | 0.513 | 28 | 4000 | 0.70 |  |  |

PCE: Polychromatic erythrocytes; NCE: Normochromatic erythrocytes; MNPCE: Micronucleated polychromatic erythrocytes, P/E: Ratio of polychromatic erythrocytes to total number of erythrocytes.

**Table S3b: Effect of AC^3®^ on bone marrow micronucleus test in Swiss Albino female mice.**

| **Dose**  **(mg/kg b.w)** | **Animal No.** | **PCE** | **NCE** | **P/E** | **MNPCE** | **Total**  **PCE** | **% MNPCE** | **No. of MNPCE/**  **Group** | **MNPCE** |
| --- | --- | --- | --- | --- | --- | --- | --- | --- | --- |
|  |  |  |  |  |  |  |  |  | **Mean**±**SD** |
| **0**  **(Vehicle Control)** | 50 | 268 | 238 | 0.530 | 1 | 4000 | 0.03 | 1 | 0.2±0.45 |
|  | 51 | 260 | 242 | 0.518 | 0 | 4000 | 0.00 |  |  |
|  | 52 | 259 | 246 | 0.513 | 0 | 4000 | 0.00 |  |  |
|  | 53 | 262 | 248 | 0.514 | 0 | 4000 | 0.00 |  |  |
|  | 54 | 253 | 248 | 0.505 | 0 | 4000 | 0.00 |  |  |
| **500** | 55 | 260 | 246 | 0.514 | 0 | 4000 | 0.00 | 4 | 0.8±0.84 |
|  | 56 | 248 | 254 | 0.494 | 2 | 4000 | 0.05 |  |  |
|  | 57 | 265 | 241 | 0.524 | 1 | 4000 | 0.03 |  |  |
|  | 58 | 245 | 258 | 0.487 | 1 | 4000 | 0.03 |  |  |
|  | 59 | 255 | 246 | 0.509 | 0 | 4000 | 0.00 |  |  |
| **1000** | 60 | 244 | 258 | 0.486 | 0 | 4000 | 0.00 | 3 | 0.6±0.89 |
|  | 61 | 254 | 252 | 0.502 | 2 | 4000 | 0.05 |  |  |
|  | 62 | 254 | 254 | 0.500 | 1 | 4000 | 0.03 |  |  |
|  | 63 | 249 | 256 | 0.493 | 0 | 4000 | 0.00 |  |  |
|  | 64 | 266 | 238 | 0.528 | 0 | 4000 | 0.00 |  |  |
| **2000** | 65 | 245 | 258 | 0.487 | 0 | 4000 | 0.00 | 2 | 0.4±0.55 |
|  | 66 | 253 | 252 | 0.501 | 0 | 4000 | 0.00 |  |  |
|  | 67 | 249 | 252 | 0.497 | 1 | 4000 | 0.03 |  |  |
|  | 68 | 261 | 248 | 0.513 | 0 | 4000 | 0.00 |  |  |
|  | 69 | 248 | 256 | 0.492 | 1 | 4000 | 0.03 |  |  |
| **50 (Positive Control- Cyclophosphamide Monohydrate)** | 70 | 253 | 248 | 0.505 | 27 | 4000 | 0.68 | 127 | 25.4±1.52 |
|  | 71 | 248 | 253 | 0.495 | 24 | 4000 | 0.60 |  |  |
|  | 72 | 242 | 262 | 0.480 | 25 | 4000 | 0.63 |  |  |
|  | 73 | 248 | 254 | 0.494 | 24 | 4000 | 0.60 |  |  |
|  | 74 | 253 | 248 | 0.505 | 27 | 4000 | 0.68 |  |  |

PCE: Polychromatic erythrocytes; NCE: Normochromatic erythrocytes; MNPCE: Micronucleated polychromatic erythrocytes, P/E: Ratio of polychromatic erythrocytes to total number of erythrocytes.
